# C-Reactive Protein as a Predictor of Complicated Acute Pancreatitis: Reality or a Myth?

**DOI:** 10.7759/cureus.19265

**Published:** 2021-11-04

**Authors:** Rami Ahmad, Khalid M Bhatti, Mooyad Ahmed, Kamran Ahmed Malik, Shafiq Rehman, Abdulmoniem Abdulgader, Ambreen Kausar, Ruben Canelo

**Affiliations:** 1 Colorectal Surgery, Royal Blackburn Hospital, Blackburn, GBR; 2 Surgery, Health Education of England, Northwest Deanery, Blackburn, GBR; 3 Surgery, Wrexham Maelor Hospital, Wrexham, GBR; 4 Hepato-Pancreatico-Biliary (HPB) Surgery, Newcastle Freeman Hospital, Newcastle, GBR; 5 Hepato-Pancreatico-Biliary (HPB) Surgery, Royal Blackburn Hospital, Blackburn, GBR; 6 Surgery, North Cumbria Integrated Care (NCIC), Carlisle, GBR

**Keywords:** modified ct severity index score (mctsi), neutrophil to lymphocyte ratio (nlr), glasgow scoring system, c-reactive protein, complicated acute pancreatitis

## Abstract

Introduction

C-reactive protein (CRP) has been reported as a predictor of the severity of acute pancreatitis (AP). However, there is conflicting evidence in the literature. The proposed cut-off values and intervals for best prediction include an absolute value of 150 at 48 hours; an absolute value of 190 at 48 hours; and the interval change in CRP of 90 at 48 hours. The current study assesses the value of CRP at different intervals and cut-offs in predicting complicated acute pancreatitis (CAP) and compares its performance against other available predictors like neutrophil to lymphocyte ratio (NLR); Glasgow scoring system and modified CT severity index (MCTSI).

Methods

Analysis of prospectively maintained data for index episodes of acute pancreatitis managed in 225 patients over a period of five years (2014-2018) was done. CAP was defined by using revised Atlanta classification and included all the AP patients with local and or systemic complications. It was used as a gold standard. Diagnostic and predictive performance of different biochemical markers and multifactorial scoring systems were determined by analyzing receiving operating curves (ROCs), the area under the curve (AUC), sensitivity, specificity, and predictive values (positive and negative).

Results

Out of 225 patients, 122 were female while 103 patients were male. CAP developed in 47 patients (20.9%) while 178 (79.1%) patients had mild AP. Overall, in-hospital mortality rate was 1.8% (n=4). ROC analysis demonstrated that CRP at admission had low discriminatory value (AUC= 0.54, p-value=0.74). CRP at 48 hours had AUC of 0.70 (p-value=0.007). At a cut-off of 150, the positive predictive value (PPV) of 150 was 30 %. The PPV of CRP at 48 hours at a cut-off of 190 was 28%. Interval change in CRP at 48 hours greater than 90 had a PPV of 26 %. Further comparison of CRP with other scoring systems like Glasgow scoring system (AUC= 0.65), NL ratio (AUC=0.54), and MCTSI was performed. Among the single predictors, although, NL ratio showed good sensitivity at a cut-off value of 4.7 (87.23%), however, its discriminatory power was negligible (AUC=0.542, p-value=0.513). The overall best performance was achieved by the MCTSI scoring system at a cut-off of 3 (AUC=0.90, sensitivity=83.33 %, specificity=100%, diagnostic accuracy=94.49%).

Conclusion

CRP measured at admission or at 48 hours has a very limited role in the prediction of CAP. Along with other scoring systems, its negative predictive value should be used to predict cases with mild AP which can help in clinical decision making for early discharge or management of such patients on ambulatory care basis. MCTSI scoring system can be used in cases with high suspicion of CAP.

## Introduction

Acute pancreatitis (AP) is a common presentation. The reported annual incidence varies from 13 to 45 per 100,000 people [1]. AP can be divided into mild AP (MAP), moderately severe AP (MSAP), and severe AP (SAP) according to the revised Atlanta classification system [2]. This classification is based on the presence or absence of local and or systemic complications. Acute pancreatitis in the absence of any local or systemic complications is labeled as MAP. MSAP is a group of patients with AP who either develop any of the local complications like acute fluid collections, acute necrotic collections, pseudocyst formation, and/ or walled-off necrosis; or who suffer from transient organ failure for less than 48 hours [2]. Patients with persistent organ failure for more than 48 hours are grouped as SAP [2]. The major concern in the management of patients with AP is the progression of MAP to its severer forms due to high morbidity and mortality associated with these conditions [3,4]. Both MSAP and SAP are sometimes classified together as complicated acute pancreatitis (CAP) and this grouping has its own pragmatic value [5,6].

The reported mortality of 30%-40 % for patients with CAP is of serious concern [7]. Due to this reason, it is not surprising that there has always been an interest in the development and/or validation of multifactorial scoring systems or single predictors that could predict patients at higher risk of progression to CAP. Ranson, Glasgow, and APACHE II, though commonly used in the past, have their own limitations due to various reasons [8]. These limitations in turn led to an interest in the development of single biochemical markers as a predictor of CAP [9]. C-reactive protein (CRP) is one of such predictors [10]. It is an acute-phase reactant and it is released from the liver in response to any inflammatory condition affecting the body [10]. The first-ever report of its utilization as a predictor of CAP was published by Mayer et al in 1984 [11]. Since then there have been conflicting reports about its value as a predictor of CAP [8,12]. Moreover, there is uncertainty about the best time to measure CRP and its best cut-off values [8, 13]. For example, Cardoso et al have reported its best utilization at 48 hours at a cut-off value between 180-190 [13]. Others have suggested a cut-off value of 150 at 48 hours as a predictor of the severity of acute pancreatitis [14]. To further complicate the situation, recently the concept of interval change in CRP of more than 90 has been proposed as a better predictor [15]. As there is no consensus on the best timing and best cut-off values for the CRP as a predictor of CRP, there is a need to compare all of the described timings and values with one another and with other commonly used predictors. 

The current study was aimed at determining the diagnostic accuracy and predictive value of CRP at different timings and cut-off values compared to other multifactorial and single predictors like neutrophil to lymphocyte ratio (NLR), Glasgow scoring system, and modified CT severity index (MCSTI) while using revised Atlanta classification as a gold standard.

## Materials and methods

The current study was conducted at North Cumbria Integrated Care (NCIC), Carlisle, United Kingdom. Ethical approval and patient consent were waived off as the current study was based on the data collected for the quality improvement project (Project No. 727). It involved a retrospective analysis of prospectively collected data for the patients admitted with AP over a period of five years (January 2014 and December 2018).

Although the database consisted of many variables, however, for the purpose of this study extracted variables included age, gender, etiology, comorbidities, CRP at admission, CRP at 48 hours, interval change in CRP at 48 hours, NLR at admission, Glasgow score, MCTSI score, and grouping based on revised Atlanta classification [2]. All the consecutive patients above the age of 18 having information about the above-mentioned CRP-related variables and final outcome as uncomplicated or complicated acute pancreatitis based on revised Atlanta classification as described in the guidelines of IAP (International Association of Pancreatology) working group and the American Pancreatic Association (APA) [1] were considered for final analysis. Mild AP included the patients without any local or systemic complications while CAP included patients suffering from MSAP and SAP as defined by the presence of any local or systemic complications. MCTSI was calculated as described by Mortele et al based on the contrast-enhanced CT scan performed at least 72 hours after the admission [2,16].

Statistical Package for the Social Sciences (SPSS), Version 20 (IBM, Corp., Chicago, IL, USA) was used for data analysis. Descriptive statistics were used to analyze different variables. Medians and modes were measured for numerical variables. For categorical variables frequencies were determined. Inferential statistics included the comparison of the two groups (uncomplicated vs complicated acute pancreatitis) for variables like age, gender, comorbidities, and etiology. The area under the curve analysis was done to compare the diagnostic performance of different multifactorial scoring systems and single predictors while considering CAP as positive and MAP as negative. Diagnostic performance was further compared using sensitivity, specificity, positive and negative predictive values, and diagnostic test accuracy. A p-value of less than 0.05 was taken as statistically significant.

## Results

Over the study period of five years, a total of 496 episodes of acute pancreatitis were managed in 401 patients. The outcomes of the whole cohort have been reported previously by our group [5]. For the current study, 225 patients meeting the inclusion criteria were included as 176 cases were missing some of the relevant data. Out of 225 patients, 122 were female while 103 patients were male. The median age was just over 65. The most common cause was biliary pancreatitis (57.8%). According to the Glasgow scoring system, 37 patients were predicted to have CAP (24.20% of the calculated) while according to the revised Atlanta classification, CAP developed in 47 patients (20.9%). Mild AP was noted in 178 (79.1%). Overall, in-hospital mortality rate was 1.8% (n = 4) (Table 1).

**Table 1:** Demographic features.

Characteristic		Value
Age	Median (range)	65.85 (18-101)
Gender	Female	122 (54.2%)
	Male	103 (45.8%)
	Total	225 (100%)
Etiology	Biliary acute pancreatitis	130 (57.8%)
	Alcohol-induced acute pancreatitis	40 (17.8%)
	Other/idiopathic acute pancreatitis	55 (24.4%)
	Total	225 (100 %)
Glasgow score	Mild pancreatitis	119 (52.9%)
	Severe pancreatitis	38 (16.9%)
	Not calculated	68 (30.2%)
	Total	225 (100%)
Severity based on modified CT severity index	Mild	92 (72.4%)
	Moderate	31 (24.4%)
	Severe	4 (3.1%)
	Not calculated (CT was not indicated)	98
	Total	225 (100%)
Revised Atlanta Classification	Mild	178 (79.1%)
	Moderately severe	29 (12.9%)
	Severe acute pancreatitis	18 (8.0%)
	Total	225 (100%)
Complicated acute pancreatitis	No	178 (79.1%)
	Yes	47 (20.9%)
	Total	225 (100%)
Mortality	No	221 (98.2%)
	Yes	4 (1.8%)
	Total	225 (100%)

Comparison of the CAP and MAP groups did not show any significant difference in the distribution by gender, age, etiology, and comorbidities (Table 2). 

**Table 2:** Comparison of complicated and mild acute pancreatitis groups by different study characteristics.

Variable	Categories	Complicated acute pancreatitis			Chi-square value	p-Value
		Yes	No	Total		
Gender	Male	22	81	103	0.02	0.50
	Female	25	97	122		
	Total	47	178	225		
Age (less than 60)	Yes	28	107	135	0.004	0.53
	No	19	71	90		
	Total	47	178	225		
Biliary pancreatitis	Yes	26	104	130	0.14	0.41
	No	21	74	95		
	Total	47	178	225		
Comorbidities	Yes	15	40	55	1.79	0.12
	No	32	138	170		
	Total	47	178	225		

**Figure 1:**
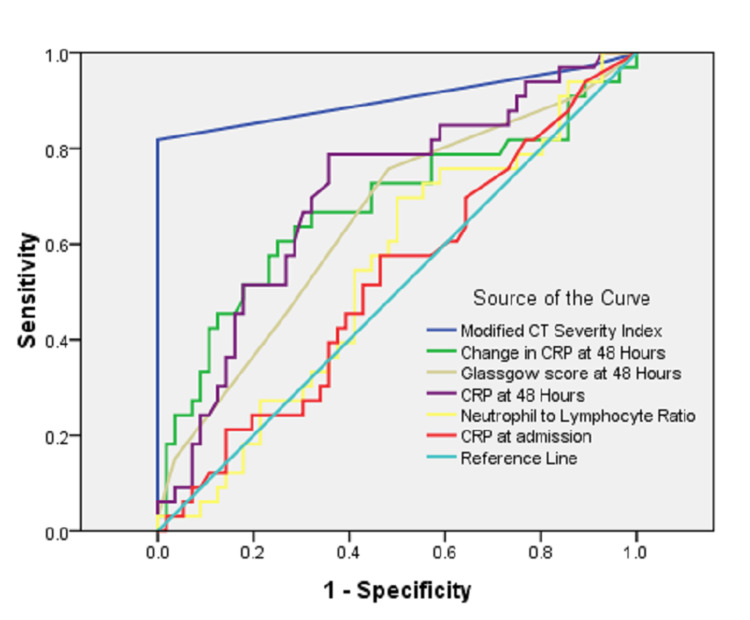
Receiving operating curve for different predictors of complicated acute pancreatitis.

Figure 1 shows the ROC analysis for different multifactorial and single predictors of complicated acute pancreatitis. 

The area under the curve for different scoring systems and predictors shows that CRP at admission, NL ratio, and Glasgow scoring system failed to discriminate CAP from MAP (Table 3). The maximum area under the curve was achieved by MCTSI followed by CRP at 48 hours and interval change in CRP at 48 hours. 

**Table 3:** Area under the curve for different predictors of complicated acute pancreatitis. ^a^Under non-parametric assumptions; ^b^computed tomogram; ^c^C-reactive protein; ^d^neutrophil to lymphocyte.

Test result variable(s)	Area	Std. error^a^	p-value	Asymptotic 95% confidence interval	
				Lower bound	Upper bound
Modified CT^b^ Severity Index Score	0.90	0.043	0.000	0.82	0.99
CRP^c^ at 48 hours	0.70	0.05	0.001	0.59	0.82
Interval change in CRP of more than 90 at 48 hours	0.67	0.06	0.006	0.55	0.80
Glasgow score at 48 hours	0.65	0.06	0.018	0.53	0.77
N/L^d^ Ratio	0.54	0.06	0.51	0.42	0.66
CRP at admission	0.52	0.06	0.74	0.40	0.64

The diagnostic and predictive performance of all the predictors is shown in Table 4. The MCTSI showed the highest sensitivity, specificity, positive predictive value (PPV), negative predictive value (NPV), and diagnostic accuracy. All of the other markers had very low sensitivity, PPV, and diagnostic accuracy. However, it is noteworthy, that the NPV of these predictors was in the range of 80%-87% (Table 4). 

**Table 4:** Comparative diagnostic and predictive performance of scoring systems.

	Cut-off value		Complicated acute pancreatitis			Sensitivity (95% CI)	Specificity (95% CI)	Predictive value (95% CI)		Accuracy (95% CI)
			Yes	No	Total			Positive	Negative	
Modified CT severity index	>3	Yes	35	0	35	83.33% (68.64-93.03)	100 % (99.75-100)	100%	92.39% (86.06-95.98)	94.49% (88.97-97.76)
		No	7	85	92					
		Total	42	85	127					
Glasgow Score	>2	Yes	16	21	37	42.11% (26.31-59.18)	82.35% (74.30-88.73)	43.24% (30.79-56.62)	81.67% (77.04-85.54)	72.61% (64.93-79.42)
		No	22	98	120					
		Total	38	119	157					
CRP at 48 hours (mg/dl)	>190	Yes	32	59	91	68.09% (52.88-80.91)	66.89% (59.42-73.72)	35.16% (28.95-41.93)	88.81%v (83.77-92.42)	67.11% (60.55-73.21)
		No	15	119	134					
		Total	47	178	225					
CRP at 48 hours (mg/dl)	>150	Yes	32	74	106	68.09% (52.88-80.91)	58.43% (50.82-65.75)	30.19% (24.97-35.98)	87.39% (81.77-91.47)	60.44% (53.73-66.88)
		No	15	104	119					
		Total	47	178	225					
CRP interval change at 48 hours (mg/dl)	>90	Yes	29	81	110	61.70% (46.38-75.49)	54.49% (46.88-61.96)	26.36% (21.35-32.07)	84.35% (78.54-88.81)	56.00% (49.25-62.59)
		No	18	97	115					
		Total	47	178	225					
Neutrophil to lymphocyte ratio	>4.7	Yes	41	148	189	87.23% (74.26-95.17)	16.85% (11.67-23.18)	21.69% (19.60-23.94)	83.33% (68.87-91.87)	31.56% (25.54-38.06)
		No	6	30	36					
		Total	47	178	225					

## Discussion

Results of the current study show that CRP at admission had very poor diagnostic accuracy and was almost nonpredictive of complicated acute pancreatitis. Similarly, at 48 hours, all the previously described cut-off values of CRP >150, CRP > 190, and interval rise > 90, failed to reach the PPV greater than 50%. Nonetheless, it is worth noting that the NPV of all of these parameters was quite good, i.e., > 80%. As these findings are quite interesting, an account of comparison with contemporary studies and the impact of these findings on clinical practice is being presented below. 

CRP at admission showed low discriminatory value (AUC= 0.54, p-value= 0.74). Similar results have been reported by Fisic et al who have reported the AUC 0.51 for the CRP measured on the first day [17]. Ke et al reported an AUC of 0.67 for day 1 CRP in the prediction of critical acute pancreatitis [18]. It is not surprising that CRP at admission was found to be unpredictable of the severity of disease as the hepatic synthesis of CRP peaks at 36-50 hours [19]. At admission, CRP is usually low if the presentation is within a few hours of the onset of the symptoms. Hence CRP at admission should not be used to predict complicated acute pancreatitis. 

In current study, CRP at 48 hours had AUC of 0.70 (p-value=0.007). Although, this is low in comparison to other studies that have reported values in the range of 0.84 to 0.90 [14,20]. However, the predictive values of CRP at 48 hours at cut-off 150 was 30 %, almost equal to that reported by Stirling et al [15]. Other studies have reported predictive values ranging from 37% to 73% [21]. The wide range in the reported values in itself is evidence that CRP at 48 hours is not reliable in predicting complicated acute pancreatitis. Moreover, the other reason for the low PPV is the lack of specificity. At 48 hours, raised levels of CRP may be due to non-pancreatitis-related complications like cholecystitis, cholangitis, urinary tract infection, or nosocomial respiratory tract infection. 

The predictive value of CRP at 48 hours at a cut-off of 190 was 28%. This is even lower than that at a cut-off value of 150, mentioned above. Our PPV was, however, not different from that reported in the literature, i.e., 31% [15]. In our case, this possibly happened due to a considerable reduction in the true positive cases from 32 to 7. With a very low PPV, CRP at 48 hours at a cut-off value of 190 is also not a good predictor of complicated acute pancreatitis. 

Lastly, interval change in CRP at 48 hours greater than 90 had a PPV of 26 % very close to 30 % reported by Stirling in a recent study [15]. Its performance was not very different from CRP at 48 hours at a cut-off value of 150 (PPV=30%) or 190 (PPV=28%). The claimed benefits of the interval change in CRP, include better sensitivity, specificity, and independence from the absolute measurement [15]. However, the current study failed to endorse these potential advantages. Even in the presence of better sensitivity and specificity, PPV of 26% would mean that only one-fourth of the patients who have CRP rise of greater than 90 may develop CAP which makes interval change in CRP a less reliable predictor. 

Studies claiming the predictive role of CRP, have reported AUC, sensitivities, and specificities. We feel that the predictive values are of the greatest relevance while determining the best predictors of complicated acute pancreatitis [22]. Although none of the predictors reached a PPV of greater than 50%, consideration of NPV for all models suggests that these scoring systems can be of great pragmatic value in screening the patients for mild disease. A similar concept has been proposed by Lankisch et al. [23]. They have suggested harmless acute pancreatitis (HAP) score aiming at the identification of patients who are less likely to develop complications [23]. We have not assessed the validity and reliability of that system, however, we feel that any patient with AP having NL ratio of less than 4.7 at admission, Glasgow score less than 2, CRP of less than 150-190 at 48 hours, and rise in CRP of less than 90 in 48 hours will have more than 80% chances of not developing the complicated acute pancreatitis. The clinical implication is that such patients can be discharged earlier or can be managed on ambulatory care basis [24]. In patients with high suspicion of the severity of the disease, a CT scan should be performed and MCTSI should be calculated. Patients with MCTSI greater than 3 should be closely monitored [25]. Discussion with the ITU team and specialist center (hub) should form the cornerstone for the management of such patients [5].

The current study was a retrospective analysis. Moreover, there are a few other limitations. Firstly, CRP at 72 hours was missing in the majority of the patients, hence was not included in the analysis. Similarly, patients with incomplete information were excluded which made the sampling purposive and convenient. This may have some impact due to selection bias and the possibility of type 1 error cannot be excluded which can affect the generalizability of the results. Finally, a CT scan was not performed on every patient, and reports were used to calculate the score. In an ideal scenario, the scoring system should have been performed by two radiologists to avoid error in the calculation of the scores [25]. With all the above-mentioned limitations, the current study has reported the value of CRP at different cut-off values and its limitations in the prediction of complicated acute pancreatitis. It has stressed the importance of describing better measures of prediction like positive and negative predictive values while reporting such studies. Moreover, it suggests the usage of different scoring systems to predict the cases that can be managed on ambulatory care basis. 

## Conclusions

CRP measured at admission or at 48 hours has a very limited role in the prediction of complicated acute pancreatitis. Along with other scoring systems, it should be used to predict cases with mild acute pancreatitis which can help in clinical decision making for early discharge or management of such patients on ambulatory care basis. Further research is needed to determine the clinical outcomes of the patients with acute pancreatitis managed using the proposed model. Due to high sensitivity, specificity, positive and negative predictive value, and diagnostic accuracy, CT scan should be used in cases with high suspicion of complicated acute pancreatitis based on holistic assessment. 
